# The impact of mixed planting of Poaceae species in the Qinghai-Tibet plateau region on forage yield, soil nutrients, and soil microbial communities

**DOI:** 10.3389/fpls.2024.1370593

**Published:** 2024-04-29

**Authors:** Sida Li, Xuemei Xiang, Zhenghai Shi, Wen-hui Liu, Guoling Liang, Yongchao Zhang, Wen Li

**Affiliations:** ^1^Key Laboratory of Superior Forage Germplasm in the Qinghai‐Tibetan plateau, Qinghai Academy of Animal Science and Veterinary Medicine, Qinghai University, Xining, China; ^2^Laboratory for Research and Utilization of Qinghai Tibet Plateau Germplasm Resources, Xining, Qinghai, China

**Keywords:** Qinghai-Tibet Plateau, mixed grasslands, *Poa pratensis L.*, *Puccinellia tenuiflora*, soil microbial communities

## Abstract

Establishing cultivated grassland in the Qinghai-Tibet Plateau region is an effective method to address the conflict between vegetation and livestock. However, the high altitude, low temperature, and arid climate in the region result in slow regeneration and susceptibility to degradation of mixed cultivation grassland containing perennial legumes and gramineous plants. Therefore, we aim to through field experiments, explore the feasibility of establishing mixed cultivation grassland of *Poaceae* species in the region by utilizing two grass species, *Poa pratensis* L. and *Puccinellia tenuiflora*. By employing a mixture of *P. pratensis* and *P. tenuiflora* to establish cultivated grassland, we observed significant changes in forage yield over time. Specifically, during the 3rd to 6th years of cultivation, the yield in the mixed grassland was higher than in monocultures. It exceeded the yield of monoculture *P. tenuiflora* by 19.38% to 29.14% and surpassed the monoculture of *P. pratensis* by 17.18% to 62.98%. Through the analysis of soil physicochemical properties and soil microbial communities in the cultivated grassland, the study suggests that the mixed grassland with *Poaceae* species can enhance soil enzyme activity and improve soil microbial communities. Consequently, this leads to increased soil nutrient levels, enhanced nitrogen fixation efficiency, and improved organic phosphorus conversion efficiency. Therefore, establishing mixed grasslands with *Poaceae* species in the Qinghai-Tibet Plateau region is deemed feasible.

## 1 Introduction

The Qinghai-Tibet Plateau covers a total area of over 2.5 million square kilometers, with an average elevation exceeding 4,000 meters. It is known as the “Roof of the World” or the “Third Pole” and plays a crucial role in global and regional climates, hydrology, and ecosystems (Deng et al., 2024). The plateau is not only the source of major Asian rivers but also one of the most biologically diverse regions in China and globally, listed as one of the 25 priority areas for biodiversity conservation by the World Wildlife Fund (Wang Q. et al., 2023; Cai et al., 2024). However, climate change and the harsh climate and infertile soils of the plateau have led to a decrease in grassland forage yield (Dong et al., 2013). As a result, to meet the economic demands in such conditions, herders may increase livestock numbers to compensate for the low efficiency of meat production (Zhu et al., 2023). But, this can lead to overgrazing and exacerbate grassland degradation in the Qinghai-Tibet Plateau (Cao et al., 2019). To address the issue of insufficient grassland forage yield in the Qinghai-Tibet Plateau, establishing perennial cultivated grasslands is considered the best strategy for improving the grassland conditions (Liu et al., 2022).

Mixed sowing grassland refers to the simultaneous cultivation of two or more crops in the same field (Suter et al., 2021). Mixed sowing grasslands are a more efficient planting model compared to monoculture grasslands. Current research indicates that mixed sowing can improve interspecies relationships, enhance grassland yield and quality, increase soil fertility, and promote the stability and diversity of soil microbial communities (Suter et al., 2021; Liu et al., 2022). Regarding soil enzyme activity, the experimental results by Zhang et al. reveal that mixed sowing of grassland leads to enhanced activities of alkaline phosphatase and urease in the soil(Zhang et al., 2022). Alkaline phosphatase primarily facilitates the hydrolysis of organic phosphorus compounds in the soil, converting them into readily available phosphate ions that can be directly absorbed and utilized by plants (Bhardwaj et al., 2023; Guo et al., 2023; Lu et al., 2023). Similarly, urease, one of the key hydrolytic enzymes in soil, catalyzes the hydrolysis of urea into NH_3_, which subsequently undergoes protonation to form NH^4+^ (Gupta et al., 2023; Zhong et al., 2024). The heightened activities of these enzymes play a significant role in promoting nitrogen and phosphorus cycling within the soil. Furthermore, the research conducted by Zhang et al. also indicates that mixed sowing grassland exhibits greater potential than monoculture grassland in enhancing microbial biomass and enriching soil microbial diversity (Zhang et al., 2022). This aspect is crucial for preserving the equilibrium of soil ecosystems. Furthermore, research conducted by Thers et al. illustrates that mixed sowing grassland can increase the activity of soil nitrate reductase (Thers et al., 2024). The activity of soil nitrate reductase affects denitrification rate and nitrogen availability for plants, serving as a pivotal link between inorganic and organic nitrogen cycling. The enhanced activity of soil nitrate reductase contributes to improved nitrogen fixation efficiency, increased total nitrogen content in the soil, and enhanced aboveground biomass in grassland (Chang et al., 2023; Xu et al., 2023). In summary, the determination of soil alkaline phosphatase, nitrate reductase, and urease activities in mixed sowing grassland can help investigate the impact of mixed sowing on soil nutrient cycling. Mixed sowing grassland can also promote the stability and diversity of soil microbial communities compared to monoculture grassland (Shu et al., 2023). Zhang et al. conducted research in sandy grassland on the Qinghai-Tibet Plateau and found that establishing long-term mixed sowing grassland can enhance soil enzyme activity and microbial biomass (Zhang et al., 2022). Additionally, other studies have demonstrated that mixed sowing grassland influences the structure of soil bacterial communities and species richness, leading to improved grassland productivity and increased organic matter content in the soil (Smith et al., 2003; Harrison and Bardgett, 2010; Ai et al., 2023; Wilschut et al., 2023). Therefore, investigating the microbial communities in cultivated grasslands helps to gain an in-depth understanding of the soil ecosystem and provides scientific evidence for enhancing crop productivity and soil quality.

Establishing mixed sowing grassland can be a key strategy to promote sustainable grassland productivity in the Qinghai-Tibet Plateau. Currently, most research is focused on the mixed planting of leguminous plants and grasses. This is primarily because leguminous plants can increase soil nitrogen content through nitrogen fixation, facilitating the transfer of nitrogen to co-planted grasses. They can also enrich the soil through the decomposition of nodules, dead leaves, and roots, thereby increasing forage production (Pires et al., 2021; Agbor et al., 2023; Ma et al., 2023). At the same time, the mixed planting of herbaceous plants and grasses can also influence the structure and function of soil microbial communities. This mixed planting can enhance the stability and adaptability of soil microbial communities and increase soil enzyme activity to promote nutrient cycling and improve soil fertility (Moreno et al., 2021; Xin and Niu, 2021). However, in the Tibetan Plateau region, the adaptability of most palatable perennial leguminous plants is compromised due to high altitude, low accumulated temperature, and arid climate. This results in lower regrowth rates and rapid degradation of perennial leguminous cultivated grassland. Given the aforementioned reasons, establishing mixed grasslands of perennial legumes and grasses in the Tibetan Plateau might not be the optimal choice. Consequently, this study aims to explore the feasibility of establishing mixed grasslands with grass species on the Tibetan Plateau. Consequently, this study aims to investigate the feasibility of establishing mixed grasslands primarily composed of different grass species on the Qinghai-Tibet Plateau through field experiments.

In 2018-2019, we used local grass species from the Qinghai-Tibet Plateau, including *Elymus sibiricus* (height approximately 80-120cm), *Festuca ovina* (height approximately 60-80cm), *Puccinellia tenuiflora* (height approximately 60cm), and *Poa pratensis* L. (height approximately 60cm), to establish a mixed sowing grassland. *P. pratensis* was chosen for its fast germination and well-developed root system. By utilizing grass species with different heights and including *P. pratensis*, we aimed to maximize sunlight utilization, reduce soil exposure time, and enhance soil erosion resistance. However, previous experimental results indicated that when three or more grass species were used for mixed sowing, interspecies competition was significant. In the second year of planting, most mixed sowing grasslands consisted of only two grass species. Interestingly, it was observed that mixed sowing grassland with *P. tenuiflora* and *P. pratensis* had significantly higher forage and seed yields compared to monoculture *P. tenuiflora* grassland and monoculture *P. pratensis* grassland. This indicated a mutualistic symbiotic relationship between the two grass species, which was not observed in other grass combinations (unpublished findings).

Based on these findings, this study hypothesizes that establishing a mixed sowing grassland with *P. pratensis* and *P. tenuiflora* can increase aboveground biomass, reduce interspecies competition, and improve the stability of the mixed sowing grassland. Additionally, this study aims to analyze the physicochemical properties and soil microbial communities in these mixed sowing grasslands to investigate the effects of mixed sowing of grass species on soil characteristics, particularly soil nutrient cycling. The objective is to explore the impact of different mixed sowing grasslands with grass species from the Poaceae family on soil characteristics.

## 2 Materials and methods

### 2.1 Experimental material and design

The experimental materials selected for this study include *Poa pratensis* var. *anceps* Gaund. cv. ‘Qinghai’ and *Puccinellia tenuiflora* cv. ‘Tongde.’ The seeds were provided by the Qinghai Academy of Animal Husbandry and Veterinary Sciences.

The experiment consists of three treatments:

1. *P. tenuiflora* monoculture grassland (*Puccinellia*)
2. *P. pratensis* monoculture grassland (*Poa*)
3. Mixed grassland of *P. pratensis* and *P. tenuiflora* (*Poa + Puccinellia*)

The experiment was sown on July 15, 2018, with each plot covering an area of 50 m² and having three replicates. The total sowing rate for each treatment was 22.5 kg·hm^-2^ with a row spacing of 30 centimeters. In the mixed sowing treatment, seeds were sown in a 1:1 ratio by seed count. Before sowing, urea was applied at a rate of 75 kg·hm^-2^, and diammonium phosphate at a rate of 150 kg·hm^-2^ was applied in the same year and the following year after the grasses regrew. Weeds were manually removed once in both the sowing year and the year after the grasses regrew. The experiment was rainfed.

### 2.2 Experimental site overview

The experimental site is located in the Perennial Forage Germplasm Resource Nursery in Xihai Town, Haibei Prefecture, Qinghai Province, China (36°59′36″N, 100°52′84″E, elevation 3,156 meters above sea level). It falls under a high-altitude continental climate characterized by long cold periods. The annual average temperature is 0.9 °C, with the highest temperature recorded at 30.5 °C and the lowest at -33.8 °C. The cumulative temperature ≥10 °C is 634.5 °C annually. The area receives abundant sunlight with strong solar radiation. The climate exhibits distinct wet and dry seasons, with rainfall and high temperatures occurring simultaneously. The annual average precipitation is 369.1 mm, and there is no absolute frost-free period. The soil type at the site is classified as black calcareous soil. Soil nutrient content is as follows: organic matter 38.35 g·kg^-1^, available nitrogen 2.58 mg·kg^-1^, available phosphorus 1.36 mg·kg^-1^, available potassium 21.69 mg·kg^-1^, and soil pH of 8.43.

### 2.3 Measurement of cultivated grassland yield

The yield of *P. pratensis* and *P. tenuiflora* was determined during the milk ripening stage in the years 2019 (2nd year), 2020 (3rd year), 2021 (4th year), 2022 (5th year), and 2023 (6th year). The experiment had 3 replicates, with three uniform 1m × 1m = 1m² quadrats selected in each plot. Each treatment was sampled 9 times. The grass in each quadrat was cut at ground level to measure the fresh weight of the pasture. In the mixed sowing treatment, the fresh weights of Poa pratensis and *P. tenuiflora* were measured separately.


$$\text{Relative yield total }(\text{RYT})=\frac{Y_{AB}}{Y_{AA}}+\frac{Y_{BA}}{Y_{BB}}$$

$$\text{Relative yield }{\left(\text{RY}\right)}^{-1}\text{xFF1A};{\text{RY}}_{A}=\frac{Y_{AB}}{Z_{AB}\times Y_{A}},{\text{RY}}_{B}=\frac{Y_{BA}}{Z_{BA}\times Y_{B}}$$

In the equation, $Y_{AB}$ represents the biomass of species A in mixed sowing, $Y_{BA}$ represents the biomass of species B in mixed sowing, $Y_{AA}$ represents the biomass of species A in monoculture, $Y_{BB}$ represents the biomass of species B in monoculture, $Z_{AB}$ represents the mixing proportion of species A in mixed sowing, and $Z_{BA}$ represents the mixing proportion of species B in mixed sowing (Fowler, 1982).

### 2.4 Soil sampling

During the milk ripening stage of *P. pratensis* and *P. tenuiflora* in August 15, 2023, rhizosphere soil samples were collected for each treatment. Plants were carefully uprooted using a spade to ensure loose soil was shaken off and adhering soil was brushed away. The adhering soil was collected as rhizosphere soil. Subsequently, each rhizosphere soil sample was sieved through a 2 mm mesh to remove plant roots and other plant material. During sampling, sterile paper was used to wipe off residues adhering to the spade, and it was disinfected before collecting the next soil sample to prevent contamination between successive treatments and maintain sample freshness. The experiment had three replicates, with sampling conducted in an “S” shape, collecting 5 replicate samples from each plot, totaling 15 samples per treatment. A portion of each soil sample was air-dried and stored at room temperature for soil enzyme activity and soil nutrient analysis. Another portion of the samples was flash-frozen in liquid nitrogen, then transported from the field to the laboratory in a portable icebox and stored at -80°C for analysis of microbial diversity.

### 2.5 Analysis of soil physicochemical properties and soil enzyme activity

Soil Moisture Content (SMC) was assessed by drying the soil in an oven at a constant mass at 105°C. Soil Bulk Density (SBD) was calculated using the weight method (Al-Shammary et al., 2018). Total Nitrogen (STN) in the soil was determined using the Kjeldahl method (Chu et al., 2017), and Total Phosphorus (STP) was measured using the molybdenum-antimony anti-adsorption spectrophotometric method (CHEN et al., 2023). The measurement of Soil Urease (SU) activity involved incubating dry 10 g of soil with a 10% urea solution at 37°C for 24 hours. The formation of ammonium was determined spectrophotometrically at 578 nm. Soil Alkaline Phosphatase (SAP) activity was determined using the phenol disulfonic acid colorimetric method (Peng and Wang, 2016). Soil nitrate reductase (SNR) activity was measured using the phenanthroline-sulfuric acid colorimetric method (Hu et al., 2014).

### 2.6 Soil DNA extraction, PCR amplification, and Illumina sequencing

Microbial DNA was extracted from 0.5 g of fresh soil four times (for a total of 2.0 g of soil) with a PowerSoil DNA Isolation Kit (MoBio Laboratories, Carlsbad, CA, USA) following the manufacturer’s protocol. The purity and quality of the genomic DNA were checked on 0.8% agarose gels. The V3-V4 hypervariable region of the bacterial 16SrRNA gene was amplified with the primers 338 F (ACTCCTACGGGAG GCAGCAG) and 806R (GGACTACHVGGGTWTCTAAT). For each soil sample, a 10-digit barcode sequence was added to the 5′ end of the forward and reverse primers (provided by Auwigene Company, Beijing). PCR was carried out on a Mastercycler Gradient Thermocycler (Eppendorf, Germany) using 50 μl reaction volumes containing 5 μl 10×Ex Taq Buffer (Mg2+ plus), 4 μl 12.5 mM dNTP Mix (each), 1.25 UEx Taq DNA polymerase, 2 μl template DNA, 200 nM barcoded primers 967 F and 1406R each, and 36.75 μl ddH2O. The cycling parameters were 94°C for 2 min, followed by 30 cycles of 94°C for 30 s, 57°C for 30 s and 72°C for 30 s, with a final extension at 72°C for 10 min. The fungal ITS region was amplified on an Eppendorf Mastercycler Gradient Thermocycler (Germany), with the primers ITS1F (5-CTTGGTCATTTAGAGGAAGTAA-3) and ITS2 (5-TGCGTTCTTCATCGATGC-3). The 5′ ends of both primers were tagged. The ultra-PAGE purified primers were ordered from Majorbio, China. The PCR mixtures were as follows: 4 μl 5× FastPfu Buffer, 1 μl each primer (5 μM), 2 μl dNTP mixture (2.5 mM), 2 μl template DNA, and 10 μlH2O. Thermocycling consisted of an initial denaturation at 95°C for 2 min, followed by 30 cycles of 95°C for 30 s, 55°C for 30 s, and 72°C for 30 s, with a final extraction at 72°C for 5 min. Three separate reactions of both bacterial and fungal samples were conducted to account for potentially heterogeneous amplification from the environmental template of each sample. PCR products were purified using the AXYGEN Gel Extraction Kit (QIAGEN) and quantified using qPCR. An equimolar mix of all three amplicon libraries was used for sequencing at Auwigene Company in Beijing, China.

### 2.7 Processing of 16S rRNA and ITS gene data

The raw sequences of bacterial and fungal reads were initially trimmed using Mothur, and sequences that had the following three criteria were kept: (1) precise primers and bar-codes; (2) quality score>30; and (3) length>200 bp. The Ribosomal Database Project (RDP) classifi;er tool (Wang, 2007) was used to classify all sequences into different taxonomic groups. Qualified reads were separated using the sample-specific barcode sequences and trimmed with Illumina Analysis Pipeline Version 2.6. Then, the dataset was analyzed using QIIME. The sequences were clustered into operational taxonomic units (OTUs) at a similarity level of 97% to generate rarefaction curves (Colwell and Coddington, 1994) and to calculate the richness and diversity indices (Maidak et al., 1994).

Bacterial Functional Group Analysis: FAPROTAX is a database based on currently cultivable bacteria, containing over 7,600 functional annotations from multiple prokaryotes. It focuses more on predicting the biogeochemical cycles of samples. In this study, FAPROTAX (http://www.ehbio.com/ImageGP/index.php/Home/Index/FAPROTAX.html) was used for functional annotation of cultivable soil bacteria.

Fungal Functional Group Analysis: The soil fungal community was functionally annotated using the FUNGuild database (http://funguild.org). Confidence levels “probable” and “highly probable” were selected for the annotations.

### 2.8 Statistical analyses

Data analysis was conducted using SPSS 22.0 to assess the significance of differences in forage yield, soil nutrients, and soil enzyme activities among different planting years of cultivated grassland. A one-way analysis of variance (ANOVA) test was employed for this purpose. Figures were generated using Origin 9.1. Taxonomic alpha diversity was calculated as the estimated community diversity by the Shannon index using the Mothur software package (v.1.30.1). Nonmetric multidimensional scaling (NMDS) was selected to illustrate the clustering of different samples and further reflect the microbial community structure, while the changes in microbial structure under intercropping patterns were referred to as microbial beta diversity. Correlations among the soil microbial compositions, soil properties, and soil enzyme activities were determined using redundancy analysis (RDA). The RDA was performed using the CANOCO 4.5 software package. The relationships between the microbial characteristics (i.e., abundance, alpha diversity and beta diversity) and the soil properties and soil enzyme activities were determined by Spearman’s correlation analysis (SPSS 19.0, SPSS Inc., Chicago, USA). The data were analyzed using a one-way analysis of variation (ANOVA) for different intercropping patterns (P < 0.05), including soil properties, soil enzyme activities and the microbial characteristics (Wang Q. et al., 2023). The difference between mean values was determined using the least significant difference (LSD) (P > 0.05) as indicated by different letters. Origin 9.1 was used to draw figures.

## 3 Results

### 3.1 Yield and interspecific competition in different types of cultivated grassland

**Figure 1:**
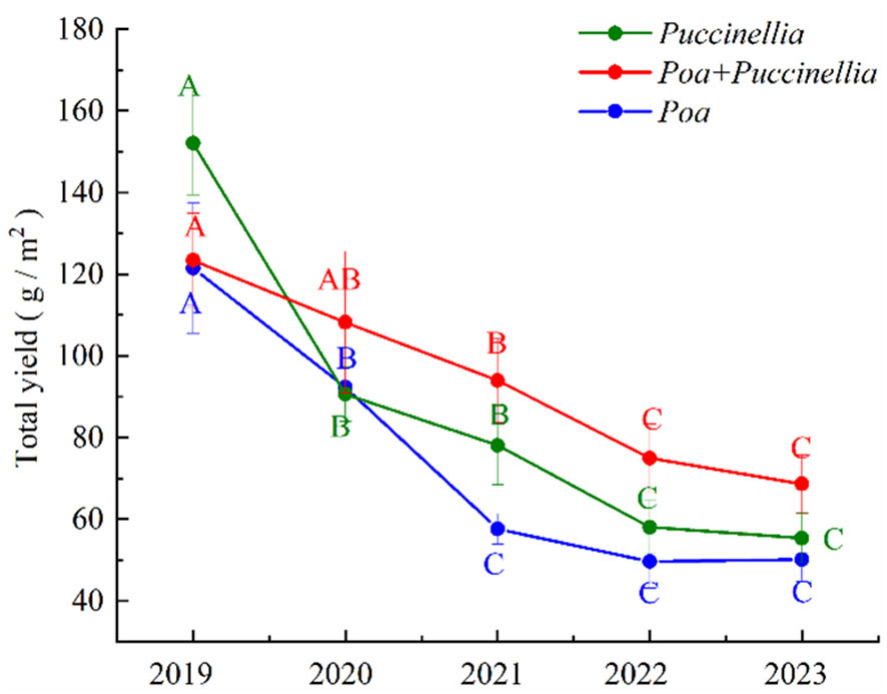
Herbage yields in different types of cultivated grassland. Different letters indicate significant differences in grass yield among cultivated grassland of different growth ages. (P<0.05).

The yield of forage in cultivated grassland is significantly influenced by the methods of sowing method and the duration of cultivation (**Supplementary Table S1**). Generally, the yield of forage in cultivated grassland shows a declining trend with the increase in years of cultivation. In the second year (2019) of cultivation, the yield of *Puccinellia* in monoculture grasslands was 152.07 g/m². As the cultivation period extended, the yield of *Puccinellia* in monoculture grasslands decreased by 40.10%, 13.81%, 25.68%, and 4.63% in the years 2020, 2021, 2022, and 2023, respectively. In the second year (2019) of cultivation, the yield of *Poa* in monoculture grasslands was 121.48 g/m². With the increase in cultivation years, the yield of *Poa* in monoculture grasslands decreased by 23.99%, 37.57%, 13.94%, and 4.63% in the years 2020, 2021, and 2022, respectively, but increased by 0.89% in 2023 (**Supplementary Figure S1**). In the second year of cultivation, the yield of mixed sowing grasslands was 123.40 g/m². With the increase in cultivation years, the yield of mixed sowing grasslands decreased by 12.21%, 13.16%, 20.21%, and 8.44% in the years 2020, 2021, 2022, and 2023, respectively. The experiments indicated that there was no significant change in the yield of forage in cultivated grassland from the fifth to the sixth year of cultivation (*P*<0.05). Moreover, the yield of *Poa + Puccinellia* in mixed sowing grasslands gradually exceeded the yield of *Poa* and *Puccinellia* with the increase in cultivation years. In the years 2019, 2021, 2022, and 2023, the yield of *Poa + Puccinellia* was higher than that of *Puccinellia* by 19.38%, 20.89%, 29.14%, and 23.97%, respectively. In 2019, the yield of *Poa + Puccinellia* was 18.86% lower than that of *Puccinellia* (**Figure 1**).

**Figure 2:**
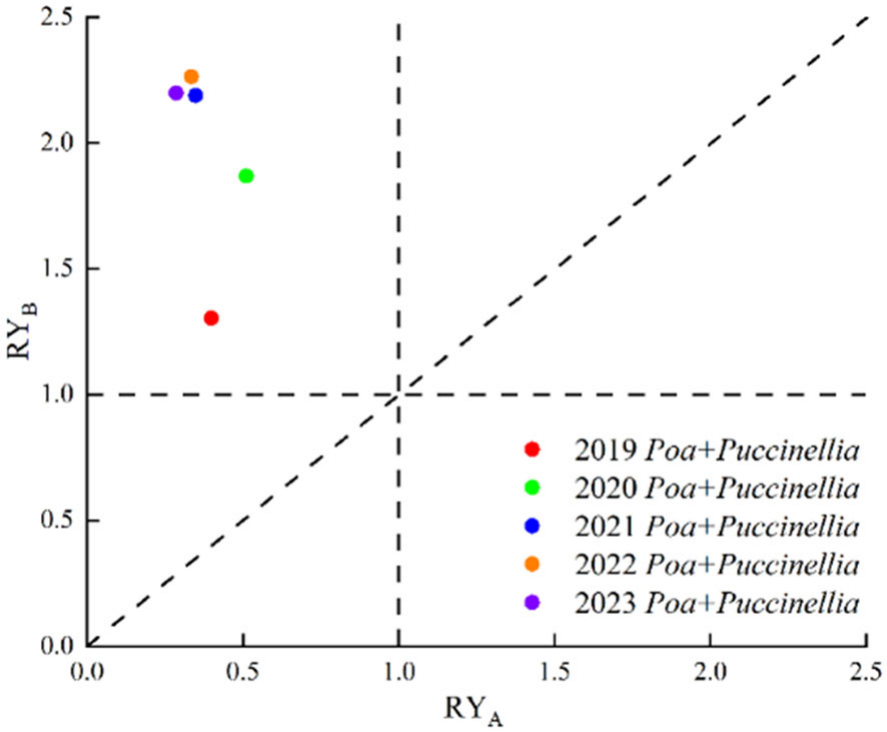
Relative yield of mixed sowing grasslands at different growth ages. RY_A_ refers to the Relative Yield of *P. pratensis*. RY_B_ indicates the Relative Yield of *P. tenuiflora*.

The interspecific relationships in mixed sowing grasslands exhibit variations. The relative biomass in mixed sowing cultivated grassland is predominantly located in the area above the RY_A_ = RY_B_ line, indicating that in these mixed grasslands, the growth of *P. pratensis* is inhibited (RY_A_ < RY_B_). From 2019 to 2023, the relative yield of *Poa + Puccinellia* consistently lies between RT_A_ < 1.0 and RT_B_ > 1.0. This suggests that in mixed sowing grasslands, the intraspecific competition of *P. pratensis* is less than its interspecific competition (RY_A_ < 1.0), whereas for *P. tenuiflora*, the interspecific competition is less than its intraspecific competition (RY_B_ > 1.0). Consequently, *P. tenuiflora* has a competitive advantage over *P. pratensis* in mixed sowing grasslands. Furthermore, from 2021 to 2023, the relative yield of *Poa + Puccinellia* remains similar (**Figure 2**).

**Figure 3:**
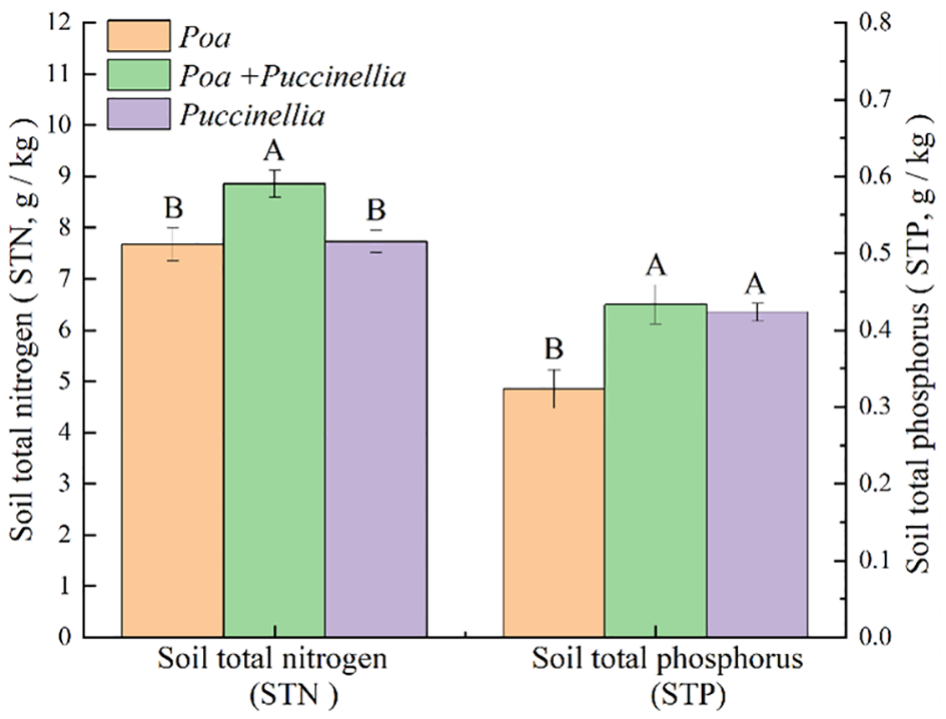
Soil nutrient content in different types of cultivated grassland in 2023. Different letters indicate significant differences. (*P*<0.05).

**Figure 4:**
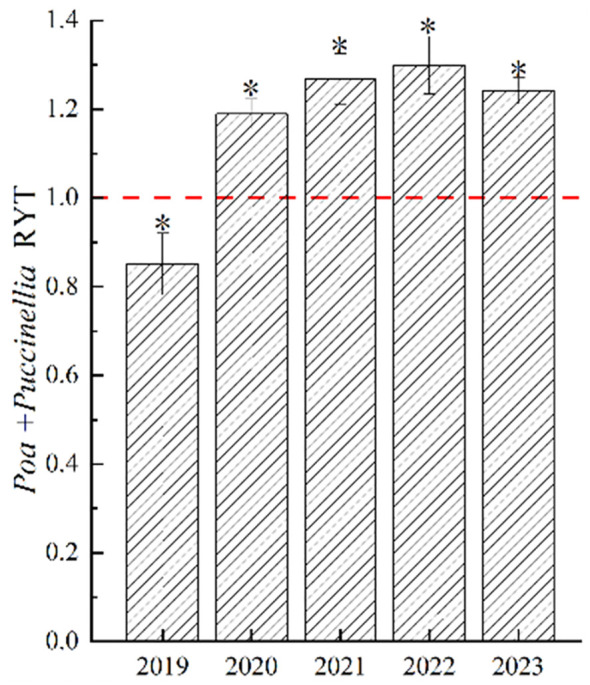
Relative yield total of mixed sowing grasslands at different growth ages. “ * “denotes significant differences in relative yields of mixed grasslands compared to 1.0 (*P<*0.01).

In 2019, the Relative Yield Total (RYT) of *Poa + Puccinellia* was significantly less than 1 (P<0.05), but with the increase in years of cultivation, the RYT of *Poa + Puccinellia* significantly exceeded 1 (P<0.05). This indicates that from 2021 to 2023, the level of competition in mixed sowing grasslands changed minimally, and the interspecific relationships within these grasslands stabilized (**Figure 3**). This suggests that in the second year of cultivation, there was intense competition in *Poa + Puccinellia* mixed sowing grasslands. However, as the cultivation period extended, the competition between *P. pratensis* and *P. tenuiflora* in these grasslands diminished. Both species, being perennial grasses and having similar plant heights, engaged in fierce competition for sunlight. Additionally, as both are grasses from the *Poa*ceae family with similar nutrient requirements, they also competed intensely for soil nutrients. However, our experiment showed that with the increase in years of cultivation, the degree of interspecific competition gradually declined and tended towards stability. We hypothesize that this is due to the alteration of the soil microbial community when *P. pratensis* and *P. tenuiflora* are mixed sown, promoting nutrient cycling and soil enzyme activity in the soil. Therefore, we conducted tests on soil enzyme activity, soil nutrients, and soil microbial diversity in the *Poa + Puccinellia* mixed sowing grasslands in the sixth year of cultivation (**Figure 4**).

### 3.2 Soil properties and soil enzyme activity in different types of cultivated grassland

The soil properties of cultivated grassland are significantly influenced by the method of mixed sowing (**Table 1**; **Supplementary Table S2**). The soil moisture content (SMC), soil bulk density (SBD), and soil porosity (SP) are notably affected, with the SMC (*P*<0.05), SBD (*P*<0.05), and SP (*P*<0.05) showing significant variation. The SMC in mixed sowing grasslands of *Poa + Puccinellia* was higher by 34.65% compared to the *Poa* monoculture grasslands and 18.33% higher than the *Puccinellia* monoculture grasslands. The SBD of the *Poa + Puccinellia* mixed sowing grasslands was 6.34% lower than that of the *Poa* monoculture grasslands and showed no significant difference (*P*>0.05) when compared to the *Puccinellia* monoculture grasslands. The SP of *Poa + Puccinellia* was 5.26% higher than the *Poa* monoculture grasslands and 7.15% lower than the *Puccinellia* monoculture grasslands, although this difference was not significant (*P*>0.05) (**Table 1**).

**Table 1:** Analysis of variance (ANOVA) for soil physical properties in intercropped grasslands.

Index	*Puccinellia*	*Poa+Puccinellia*	*Poa*	F	P
soil moisture content (SMC)	14.72 ± 1.45c	19.82 ± 0.54a	16.75 ± 0.18b	24.549	0.001
soil bulk density (SBD)	1.33 ± 0.03b	1.32 ± 0.04b	1.41 ± 0.01a	8.111	0.020
soil porosity (SP)	42.66 ± 1.17c	39.61 ± 0.29b	37.63 ± 0.33a	37.335	<0.001

Different letters indicate significant differences. (*P*<0.05). NS means the difference is not significant.

Regarding soil nutrients, the soil total nitrogen (STN) in the mixed sowing grasslands of *Poa + Puccinellia* was 4.61% higher compared to the *Poa* monoculture grasslands and 7.27% higher than the *Puccinellia* monoculture grasslands. The STN in *Poa + Puccinellia* mixed sowing grasslands was 4.09% higher than in *Puccinellia* monoculture grasslands, but this difference was not statistically significant (*P*>0.05) when compared to the *Poa* monoculture grasslands (**Supplementary Table S2**; **Figure 3**).

**Figure 5:**
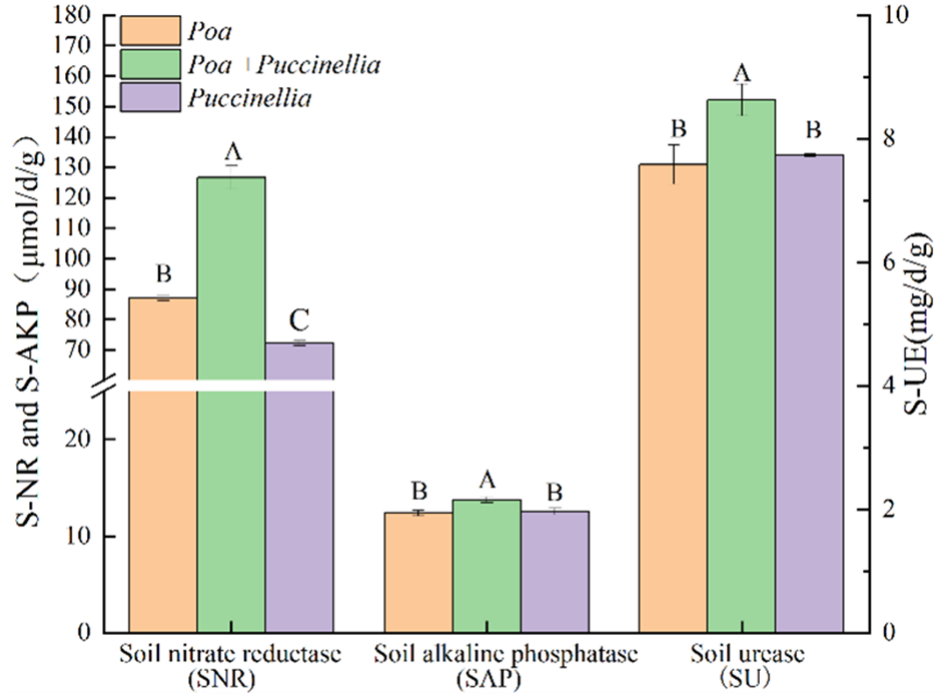
Soil enzyme activity in different types of cultivated grassland in 2023. Different letters indicate significant differences. (*P*<0.05).

In terms of soil enzyme activity, the soil nitrate reductase (SNR), soil acid phosphatase (SAP), and soil urease (SU) activities in *Poa + Puccinellia* mixed sowing grasslands were significantly higher than in *Poa* and *Puccinellia* monoculture grasslands. Specifically, the SNR in *Poa + Puccinellia* mixed sowing grasslands was 45.40% higher than in *Puccinellia* monoculture grasslands and 75.44% higher than in *Poa* monoculture grasslands. The SAP in *Poa + Puccinellia* mixed sowing grasslands was 10.91% higher compared to *Puccinellia* monoculture grasslands and 9.65% higher than *Poa* monoculture grasslands. Similarly, the SU in *Poa + Puccinellia* mixed sowing grasslands was 13.79% higher than in *Puccinellia* monoculture grasslands and 11.61% higher than in *Poa* monoculture grasslands (**Supplementary Table S2**; **Figure 5**).

### 3.3 Soil microbial community structure in different types of cultivated grassland

The Goods Coverage indices for bacterial and fungal communities in the soils of Puccinellia, Poa + Puccinellia, and Poa grasslands all reached 1. This indicates that the sequencing results comprehensively represent the true state of microbial diversity in the rhizosphere of these grasslands. Experimental results show that compared to monoculture Puccinellia and Poa grasslands, the Poa + Puccinellia mixed grassland has no significant effect on the Chao1 index, ACE, sobs, and Pielou index of bacterial communities (P>0.05) (see **Supplementary Table S3**), but has significant effects on the Shannon and Simpson indices (**Table 2**). This suggests that the Poa + Puccinellia mixed grassland has a minimal impact on the abundance and evenness of bacterial communities but significantly increases the richness of bacterial communities (P<0.05). For fungal communities, the Poa + Puccinellia mixed grassland reduces the Chao1 index and ACE but has no significant effect on sobs, while significantly increasing the Pielou and Shannon indices (P<0.05). This indicates that the Poa + Puccinellia mixed grassland has a minimal impact on the abundance of fungal communities but significantly enhances the evenness and richness of fungal communities (P<0.05) (**Table 2**).

**Table 2:** Alpha diversity of soil microbial communities in different types of artificial grasslands.

		*Puccinellia*	*Poa+Puccinellia*	*Poa*
Bacterial	sobs	4323 ± 41.94 NS	4532 ± 201.65 NS	4306 ± 74.27 NS
	chao	4530.96 ± 41.57 NS	4737.46 ± 195.01 NS	4508.12 ± 63.36 NS
	ACE	4868.7 ± 37.21 NS	5087.97 ± 191.78 NS	4847.9 ± 50.82 NS
	shannon	9.64 ± 0.04 b	9.67 ± 0.03 ab	9.75 ± 0.05 a
	simpson	0.99 ± 0.000 NS	0.99 ± 0.000 NS	0.99 ± 0.001 NS
	pielou	0.80 ± 0.00 NS	0.80 ± 0.00 NS	0.81 ± 0.00 NS
Fungal	sobs	977 ± 17.21 NS	934 ± 29.82 NS	968 ± 30.45 NS
	chao	1067.49 ± 22.45 ab	1025.50 ± 37.38 b	1093.93 ± 32.54 a
	ACE	1077.82 ± 17.44 ab	1037.50 ± 31.12 b	1099.74 ± 31.74 a
	shannon	6.76 ± 0.03 b	6.89 ± 0.02 a	6.71 ± 0.01 b
	simpson	0.98 ± 0.001 a	0.98 ± 0.001 a	0.97 ± 0.000 b
	pielou	0.68 ± 0.00 b	0.70 ± 0.00 a	0.68 ± 0.00 b

Different letters indicate significant differences. (*P*<0.05).

**Figure 6:**
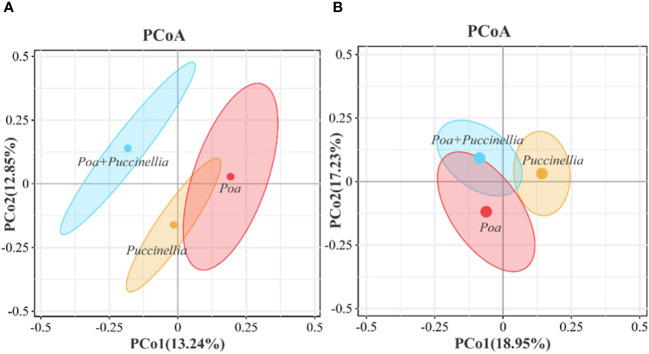
Beta diversity of soil microbial communities in different types of cultivated grassland in 2023. **(A)** represents the bacterial community, while **(B)** represents the fungal community.

**Figure 7:**
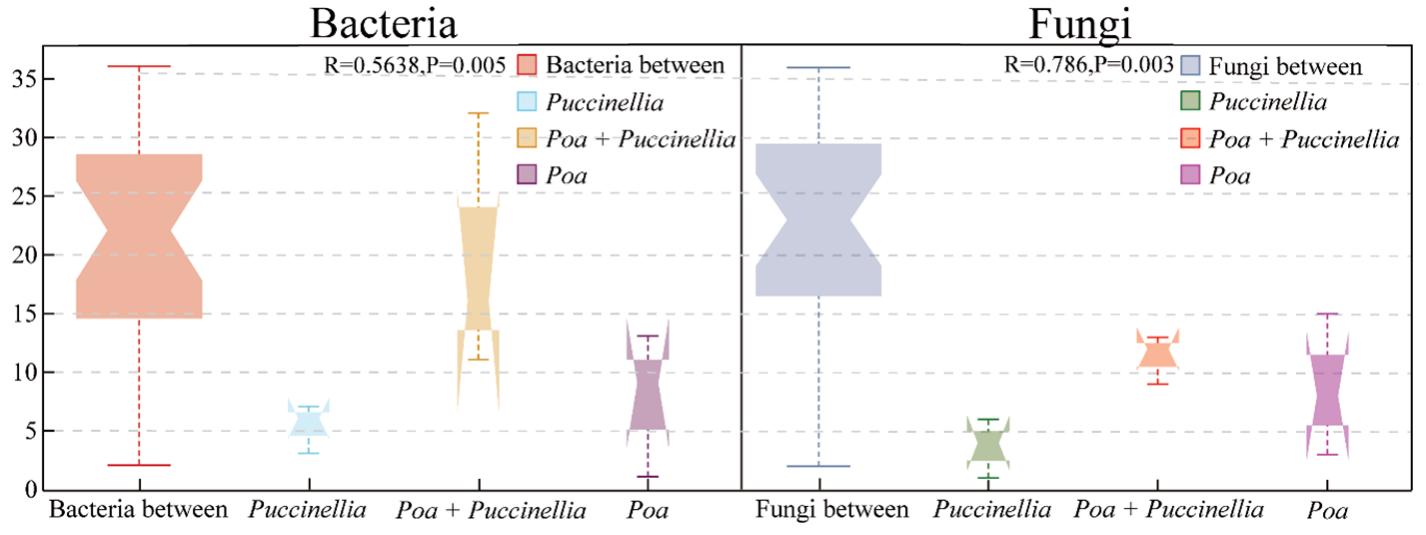
ANOSIM analysis of beta diversity based on soil microbial communities in 2023.

Principal Coordinates Analysis (PCoA) and Analysis of Similarities (ANOSIM) were employed to assess the beta diversity of microbial communities (**Figures 6**, **7**). Principal coordinate 1 accounted for 13.24% and 18.95% of the total variation in the two groups, respectively, while principal coordinate 2 accounted for 12.85% and 17.23%. Moreover, ANOSIM revealed significant differences in the microbial abundance of soil bacteria and fungi among *Puccinellia*, *Poa + Puccinellia*, and *Poa* grasslands (R=0.5638, *P*=0.005; R=0.786, *P*=0.003) (**Figures 6**, **7**).

**Figure 8:**
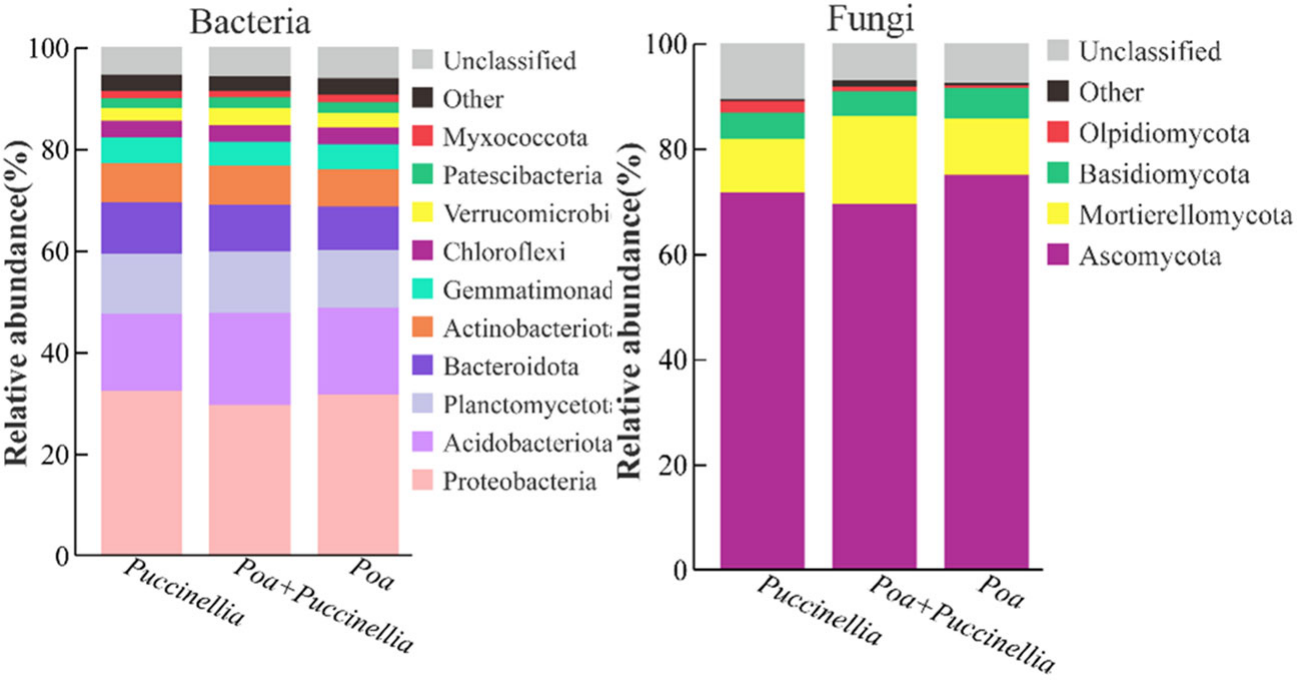
Changes in microbial (bacterial and fungal) taxonomic composition at the phylum level under mixed cropping patterns. The abundance of each taxon was calculated as the percentage of sequences per gradient for a given microbial group.

**Figure 9:**
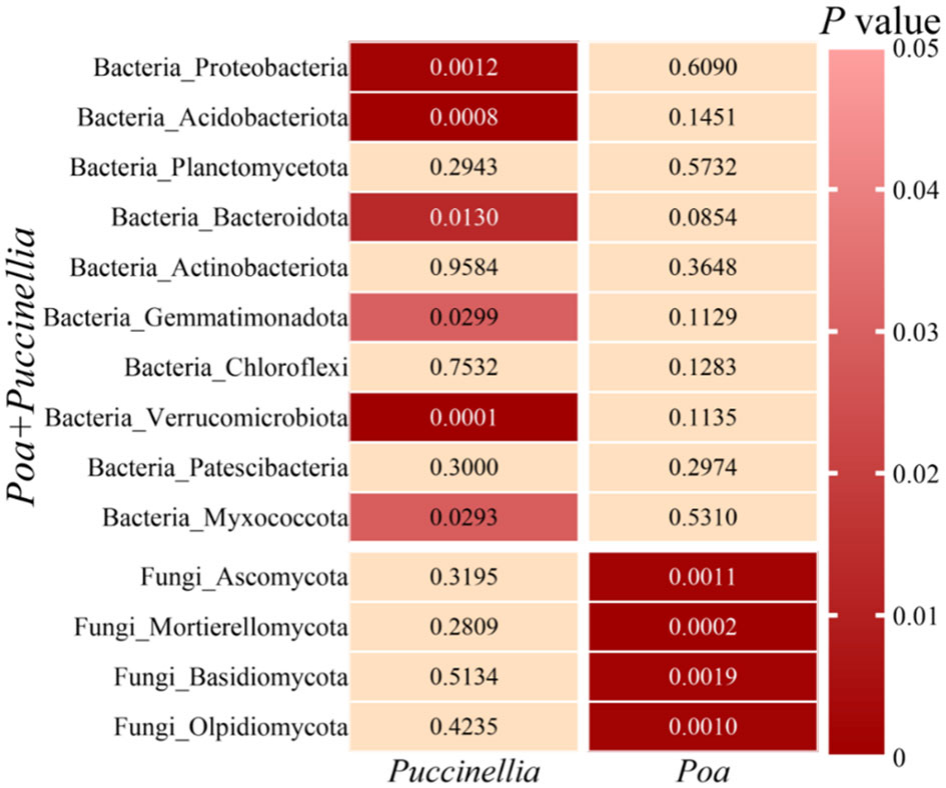
Differences in microbial composition at the phylum level between mixed cropping grasslands and monoculture grasslands.

In all grasslands, the dominant bacterial phyla with abundance greater than 1% were consistent, including *Proteobacteria*, *Acidobacteriota*, *Planctomycetota*, *Bacteroidota*, *Actinobacteriota*, *Gemmatimonadota*, *Chloroflexi*, *Verrucomicrobiota*, *Patescibacteria*, and *Myxococcota* (**Figure 8**). Similarly, the dominant fungal phyla with abundance greater than 1% were *Ascomycota*, *Mortierellomycota*, *Basidiomycota*, and *Olpidiomycota* (**Figure 8**; **Supplementary Table S4**). The mixed sowing of *Poa + Puccinellia* significantly affected the abundance of bacterial phyla such as *Proteobacteria*, *Acidobacteriota*, *Bacteroidota*, *Gemmatimonadota*, *Verrucomicrobiota*, and *Myxococcota* compared to *Puccinellia* monoculture grasslands (*P*<0.05), but the effect was not significant compared to *Poa* monoculture grasslands (*P*>0.05). However, compared to *Poa* monoculture grasslands, the mixed sowing of *Poa + Puccinellia* significantly affected the abundance of fungal phyla such as *Ascomycota*, *Mortierellomycota*, *Basidiomycota*, and *Olpidiomycota* (*P*<0.05), while the effect was not significant compared to *Puccinellia* monoculture grasslands (*P*>0.05) (**Figure 9**; **Supplementary Table S4**).

**Figure 10:**
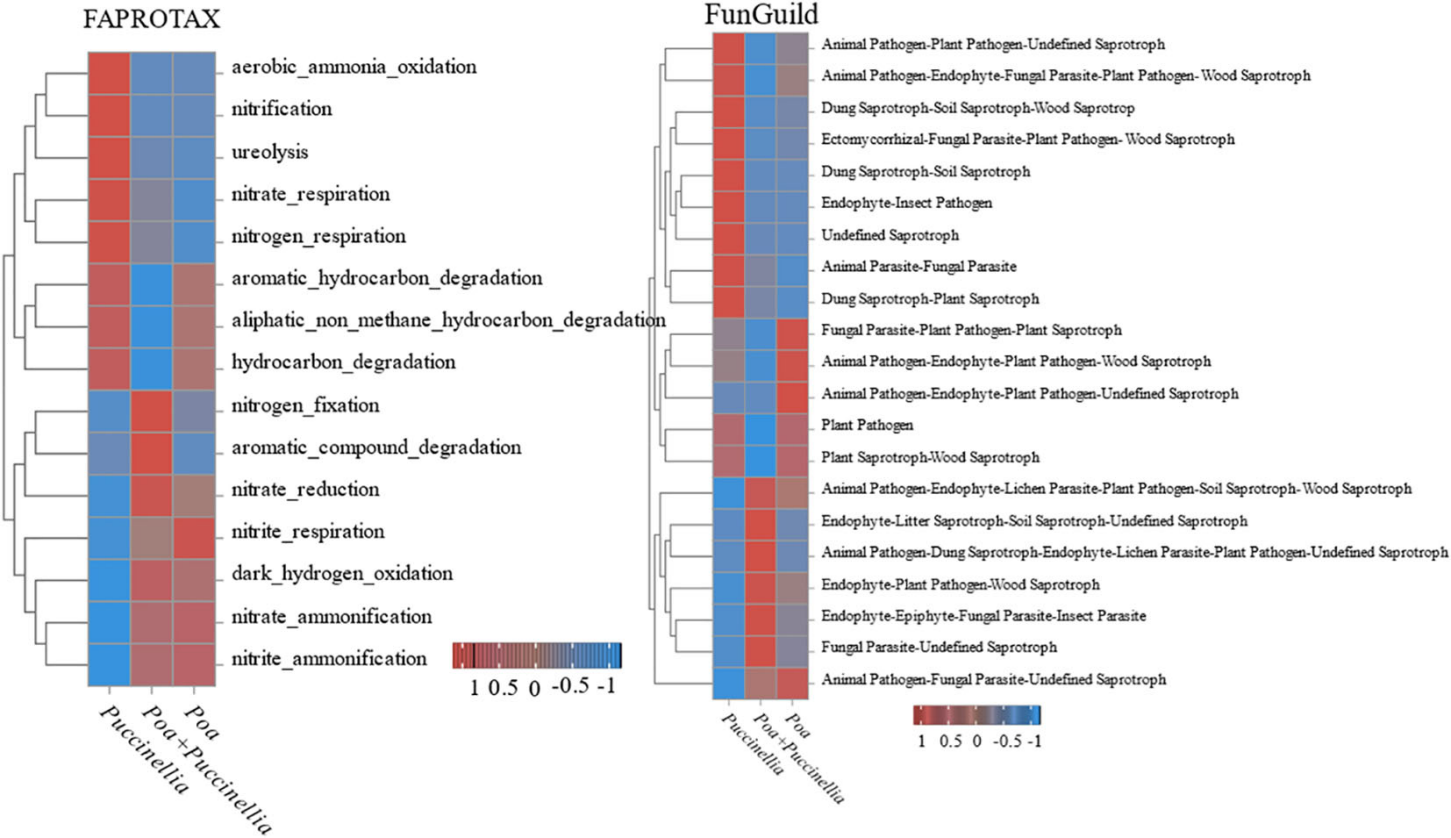
Changes in the composition of microbial functional groups.

Functional Annotation of Prokaryotic Taxa (FAPROTAX) was used to annotate the functional capabilities of bacteria in cultivated grassland. The results indicated that mixed sowing grasslands increased he gene abundance for nitrogen fixation and aromatic compound degradation. However, there was a reduction in gene abundance for aromatic hydrocarbon degradation, aliphatic non-methane hydrocarbon degradation, and hydrocarbon degradation overall (**Figure 10**).

Furthermore, FunGuild was employed for the functional annotation of fungi in cultivated grassland. The results showed that mixed sowing grasslands increased the gene abundance for various functional groups, including Animal Pathogen-Endophyte-Lichen Parasite-Plant Pathogen-Soil Saprotroph-Wood Saprotroph, Endophyte-Litter Saprotroph-Soil Saprotroph-Undefined Saprotroph, Animal Pathogen-Dung Saprotroph-Endophyte-Lichen Parasite-Plant Pathogen-Undefined Saprotroph, Endophyte-Plant Pathogen-Wood Saprotroph, Endophyte-Epiphyte-Fungal Parasite-Insect Parasite, and Fungal Parasite-Undefined Saprotroph. Conversely, there was a decrease in the gene abundance for Plant Pathogen, Plant Saprotroph-Wood Saprotroph, Fungal Parasite-Plant Pathogen-Plant Saprotroph, Animal Pathogen-Endophyte-Plant Pathogen-Wood Saprotroph, Animal Pathogen-Endophyte-Fungal Parasite-Plant Pathogen-Wood Saprotroph, and Animal Pathogen-Plant Pathogen-Undefined Saprotroph (**Figure 10**).

**Figure 11:**
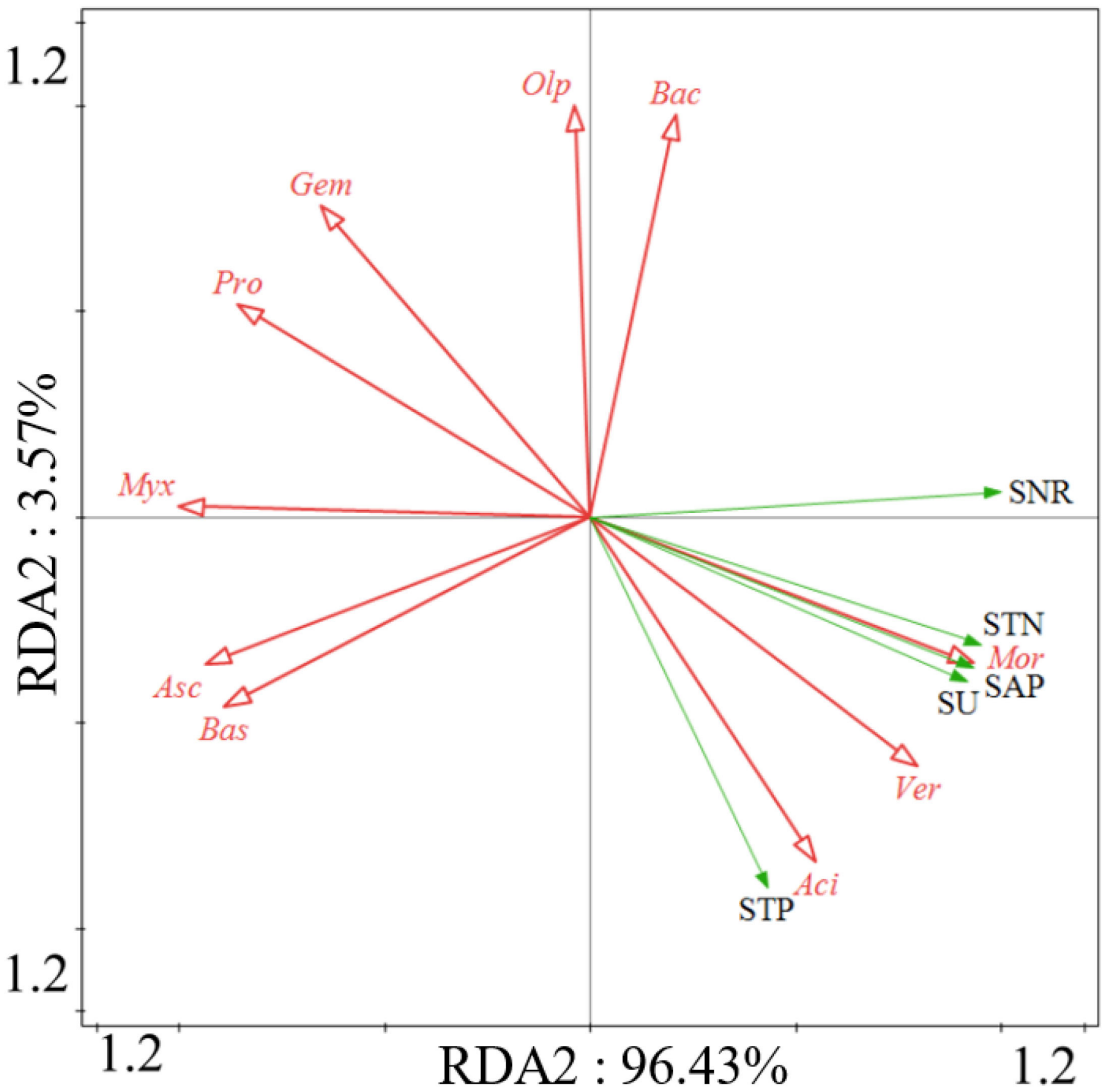
Ordination plots of the results from the redundancy analysis (RDA) to identify the relationships among the microbial (bacterial and fungal) taxa (red arrows) and the soil properties and enzyme activities (green arrows) at the phylum level. Bacterial taxa: *Bacteroidota* (*Bac*), *Verrucomicrobiota* (*Ver*), *Acidobacteriota* (*Aci*), *Myxococcota* (*Myx*), *Proteobacteria* (*Pro*), *Gemmatimonadota* (*Gem*). Fungal taxa: *Mortierellomycota* (*Mor*), *Basidiomycota* (*Bas*), *Ascomycota* (*Asc*), *Olpidiomycota* (*Olp*).

Redundancy analysis (RDA) demonstrated that soil nutrients and enzyme activities have different responses to variations in bacterial and fungal phyla. There is a significant positive correlation between soil total nitrogen (STN) and soil total phosphorus (STP), as well as soil enzyme activities such as soil nitrate reductase (SNR), soil acid phosphatase (SAP), and soil urease (SU) with the variation in bacterial *Acidobacteriota*, *Verrucomicrobiota*, and the fungal phylum *Mortierellomycota* (**Figure 11**).

**Figure 12:**
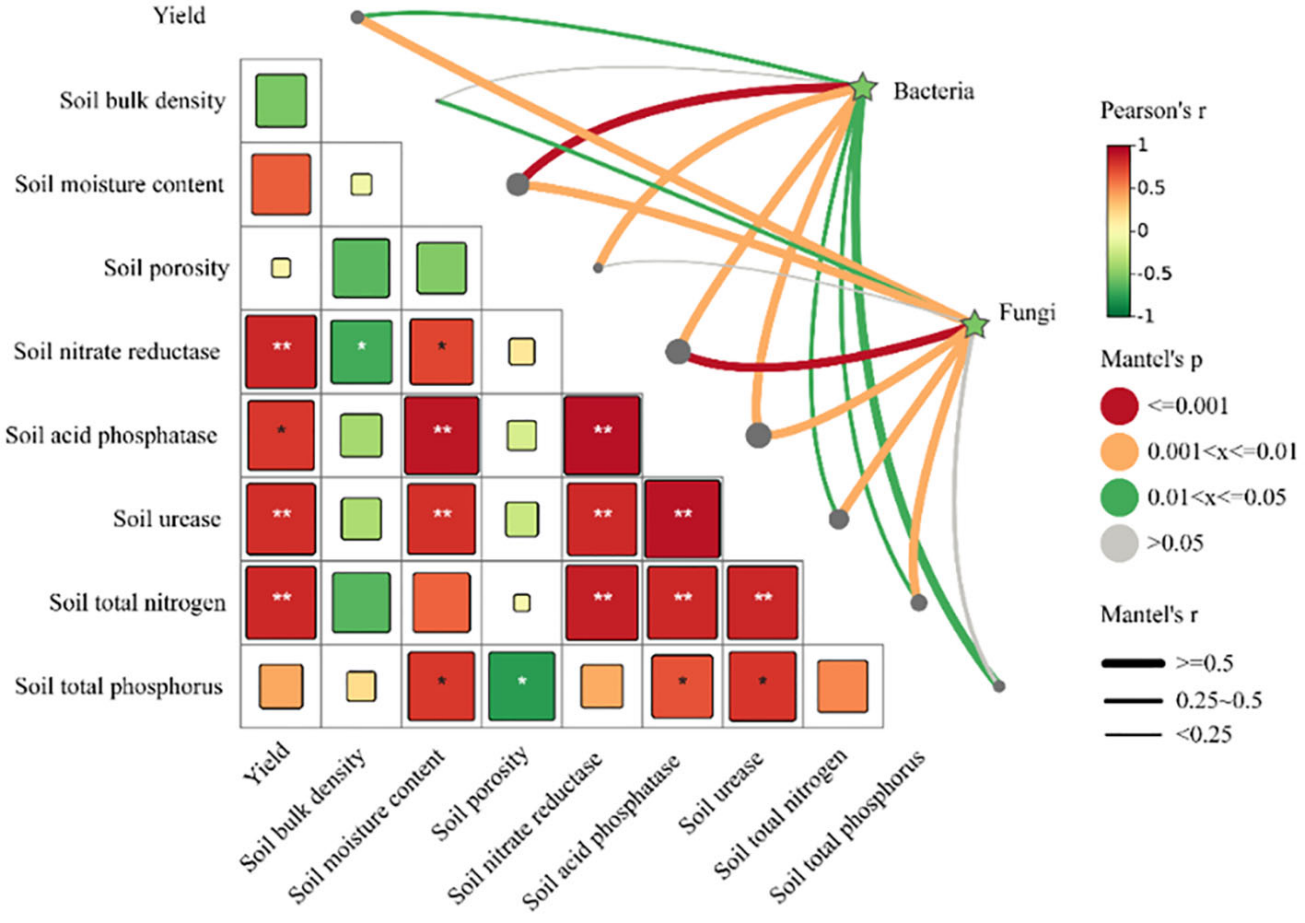
Study on the correlation between yield, soil properties, and microbial community composition. Mantel test, based on Bray-Curtis distance, shows correlations between bacteria, fungi, and both yield and soil properties. The line width corresponds to the local Mantel’s r statistic, and the line color indicates statistical significance across 999 permutations. Pairwise comparisons of environmental factors are also displayed, with color gradients representing Pearson correlation coefficients. These factors are grouped into four categories based on the attributes of the data investigated. Asterisks indicate statistically significant differences (*P < 0.05; ** *P* < 0.01).

Pearson’s correlation analysis indicates that STN, SU, SNR, SAP are significantly correlated with the yield of cultivated grassland (*P*<0.05). Using the Mantel test, it was found that SMC, SP, SNR, SAP are significantly correlated with bacterial communities, and yield, SMC, SNR, SAP, SU, STN are significantly correlated with fungal communities (**Figure 12**).

## 4 Discussion

### 4.1 Mixed sowing reduces interspecific competition and increases forage yield in cultivated grassland

Previous studies have shown that high seeding density in Cultivated grasslands leads to intense competition and plant mortality. However, as cultivation time increases and plant density decreases, competition among plants in cultivated grasslands stabilizes (Schippers and Kropff, 2001; Partzsch, 2011). Our research demonstrates that the Relative Yield of *P. tenuiflora* and *P. pratensis* in mixed sowing grasslands tends to stabilize with increasing cultivation years. Furthermore, the forage yield of all treatments shows a decreasing trend over time, but stabilizes from 2021 (the 4th year of cultivation) to 2023 (the 6th year of cultivation). These findings are consistent with previous research, suggesting that with increasing cultivation time, all treatments in cultivated grasslands reach a stable competitive state by 2021 (the 4th year of cultivation). Furthermore, our experiment indicates that in 2021 (the 4th year of cultivation), the forage yield in cultivated grasslands stabilizes. Additionally, the forage yield of mixed sowing grasslands of *P. tenuiflora* and *P. pratensis* is significantly higher than that of monoculture grasslands of *P. tenuiflora* and *P. pratensis*. Therefore, mixed sowing of *P. tenuiflora* and *P. pratensis* can increase the forage yield of grasslands.

Numerous studies have demonstrated that greater niche overlap leads to more intense interspecific competition for limited resources, resulting in reduced community stability and lower yields (Baillard et al., 2021; Reiss and Drinkwater, 2022; Glowacki et al., 2023). Our study findings reveal that in 2019 (the second year of cultivation), the forage yield of Poa + Puccinellia mixed sowing grasslands was lower than that of monoculture grasslands, with a significantly lower Relative Yield Total (RYT<1.0, P<0.05). This could be attributed to the excessive planting density during the early growth stages and the intense competition for photosynthesis and nutrient uptake between *P. tenuiflora* and *P. pratensis*, both of which belong to the *Poaceae* family and are classified as bottom leaf grasses. These grasses have similar plant heights and nutrient requirements, resulting in fierce competition for light and nutrients (Golińska et al., 2023; Skuodienė et al., 2023). However, as the mixed sowing grasslands grew, the RYT increased to greater than 1.0 (P<0.05), indicating an increase in interspecific compatibility and a decrease in competition. This change can be attributed to the growth of species in the mixed sowing grassland over time, a decrease in planting density, and an increase in available ecological factors, resulting in a reduction in ecological niche overlap between competitors and a reduction in interspecies competition (Niu et al., 2009). Therefore, the forage yield in the mixed sowing grassland gradually surpasses that of monoculture grasslands of *P. tenuiflora* and *P. pratensis*.

### 4.2 Mixed sowing enhances soil enzyme activity and soil nutrients, reducing interspecific competition in mixed sowing grasslands

Previous studies have shown that when the activity of soil alkaline phosphatase is enhanced, the rate of conversion of organic phosphorus to inorganic phosphorus in the soil accelerates, increasing the content of available phosphorus (Kuzmanović et al., 2024; Rocabruna et al., 2024). An increase in the activity of soil nitrate reductase can affect the rate of soil denitrification and the amount of nitrogen available to plants (Li et al., 2024; Paludo et al., 2024). Soil urease catalyzes the hydrolysis of urea into NH_3_, which is further converted to NH^4+^ through proton exchange (Qin et al., 2010; Pinto et al., 2014; Chen et al., 2021; Duff et al., 2022). Consistent with previous studies, our experiment demonstrated a significant positive correlation between the total nitrogen content of mixed sowing grasslands and the activities of nitrate reductase and urease. Additionally, we observed a significant positive correlation between the total phosphorus content and the activity of soil alkaline phosphatase. This indicates that the mixed sowing grassland of *P. tenuiflora* and *P. pratensis* can promote soil nutrient cycling by increasing the enzymatic activity of soil alkaline phosphatase, soil nitrate reductase, and soil urease.

A plethora of studies have consistently demonstrated that mixed sowing grasslands can enhance soil nutrient availability by decomposing plant litter into mineralized nutrients, thereby increasing the nitrogen and phosphorus content in the soil (Horrocks et al., 2016; Husse et al., 2017; Sollenberger et al., 2019). Additionally, the secretion of organic acids and enzymes by the root system of mixed sowing grasslands can facilitate the release of locked nitrogen and phosphorus in the soil, ultimately improving their availability (Li et al., 2023; Wang Z. et al., 2023). Moreover, mixed sowing grasslands can contribute to the enhancement of soil nitrogen-fixing capabilities by improving the soil microbial community (Philippot et al., 2023). These pathways collectively contribute to the increase in soil total nitrogen and total phosphorus content. Consistent with prior research findings, the soil total nitrogen and total phosphorus content in *P. tenuiflora* and *P. pratensis* mixed sowing grasslands were significantly higher compared to monoculture grasslands. This suggests that cultivated grasslands established through the mixed sowing of *P. tenuiflora* and *P. pratensis* can effectively enhance the total nitrogen and total phosphorus content in the soil.

However, previous studies have indicated that increasing or altering the ecological factors utilized by species can reduce niche overlap among competing species, thereby mitigating interspecific competition and benefiting the stability of competitive communities (Morales et al., 2023). Our study shows that the levels of soil total nitrogen (STN) and soil total phosphorus (STP) in mixed sowing grasslands are significantly higher than in monoculture grasslands of *P. tenuiflora* and *P. pratensis* (P<0.05). By 2023 (the sixth year of cultivation), the forage yield of mixed sowing grasslands had surpassed that of the monoculture *P. tenuiflora* and *P. pratensis* grasslands, with a Relative Yield Total (RYT) greater than 1.0 (P<0.05). This can be attributed to the higher levels of STN and STP in the soil of Poa + Puccinellia mixed sowing grasslands compared to monoculture grasslands. These elevated nutrient levels help promote cell division and leaf expansion, increasing leaf area, beneficial for capturing light energy and enhancing photosynthesis. Additionally, it enables plants to develop more extensive root systems, advantageous for maximizing the absorption of soil moisture and minerals, leading to niche differentiation between the two plants and reducing interspecific competition in the mixed sowing grasslands.

### 4.3 The impact of mixed sowing on soil microbial communities

The microbial diversity index, a key indicator of species richness in soil microbial communities, suggests that higher indices correlate with richer microbial diversity, more complex ecosystems, and greater functional stability (Deng, 2012; Bastida et al., 2021; Osburn et al., 2023). Our study shows that mixed sowing significantly increases soil bacterial alpha diversity (*P*<0.05). indicating that mixed sowing enhances bacterial diversity, ecosystem complexity, and functional stability compared to monoculture grasslands (Diakhaté et al., 2016).

Our investigation reveals that mixed sowing significantly modulates the functional gene composition in soil bacterial communities, notably enhancing the frequency of genes implicated in the degradation of aromatic compounds and in nitrogen fixation processes. Aromatic compounds are ubiquitously present in nature, including in all biological entities, and their degradation predominantly occurs through microbial processes that transform complex organic matter into forms utilizable by microbes and plants (Shen et al., 2012; Shahsavari et al., 2019; Singh and Kumar, 2019). The increased abundance of genes associated with aromatic compound degradation in the bacterial communities of mixed sowing grasslands indicates that mixed sowing enhances the activity of soil bacteria in decomposing aromatic compounds, thereby improving soil fertility and productivity (Li et al., 2020; Wang Z. et al., 2023). Furthermore, the increased abundance of soil nitrogen fixation genes can enhance the soil’s nitrogen fixation capacity, increase soil nitrogen levels, and thus alleviate nutrient competition in mixed sowing grasslands (Dai et al., 2018).

Our research findings indicate that mixed sowing grasslands significantly decrease the abundance of the *Ascomycota* phylum within the fungal community and considerably increase the abundance of the *Mortierellales* phylum. The *Ascomycota* phylum is a critical driver of carbon and nitrogen cycling in arid ecosystems (Wang Z. et al., 2022). Previous studies have shown that long-term application of nitrogen fertilizers promotes the growth of copiotrophic microorganisms in the soil while inhibiting oligotrophic microorganisms (Challacombe et al., 2019). This aligns with our study’s results, where mixed sowing of Poa + Puccinellia grasslands increases soil total nitrogen content, leading to a reduction in the abundance of the *Ascomycota* phylum.

The *Mortierellales* phylum plays a significant role in soil nutrient cycling (Davies et al., 2010). Our study indicates a positive correlation between the *Mortierellales* phylum and soil STN, SAP, SU, STP, aligning with prior research findings. Additionally, through the Mantel test, we demonstrated a significant positive correlation between yield in mixed sowing grasslands and soil fungal community structure. Therefore, our study suggests that mixed sowing can enhance soil nutrient transformation efficiency by improving the soil fungal community, thereby increasing soil nutrients and reducing interspecific competition in mixed sowing grasslands.

## 5 Conclusion

Our study demonstrates that in the Qinghai-Tibet Plateau region, mixed sowing of *P. pratensis* and *P. tenuiflora* can enhance the forage yield, soil total nitrogen and total phosphorus content, and soil enzyme activity in cultivated grasslands. Additionally, the mixed sowing of *P. pratensis* and *P. tenuiflora* effectively promotes the soil microbial community and enhances its stability. The results of our research indicate that mixed sowing of *P. pratensis* and *P. tenuiflora* can enhance the activities of soil urease, alkaline phosphatase, and nitrate reductase, as well as increase the gene abundance of *Mortierellales* fungi and the nitrogen-fixing functional genes in bacteria. This contributes to the improved efficiency of organic phosphorus conversion to inorganic phosphorus and soil nitrogen fixation efficiency. Therefore, establishing mixed sowing grasslands of *Poaceae* species in the Qinghai-Tibet Plateau region is considered highly feasible. However, there are still some issues in current research, such as high sowing density, limited experimental sites, and a lack of investigation into the species diversity of cultivated grasslands. Thus, further studies can optimize sowing density, conduct multi-site validation, strengthen investigations into cultivated species diversity, and delve into the impact of cultivated grasslands on soil ecosystem functions. This will further refine the cultivation techniques for mixed sowing grasslands of *Poaceae* species, promoting increased grassland productivity and ecosystem stability.

## Data availability statement

The original contributions presented in the study are included in the article/**Supplementary Material**. Further inquiries can be directed to the corresponding author.

## Author contributions

SL: Conceptualization, Formal analysis, Investigation, Methodology, Software, Writing – original draft, Writing – review & editing. XX: Formal analysis, Investigation, Visualization, Writing – review & editing. ZS: Investigation, Validation, Writing – review & editing. W-HL: Conceptualization, Data curation, Formal analysis, Funding acquisition, Investigation, Methodology, Project administration, Resources, Software, Supervision, Validation, Visualization, Writing – original draft, Writing – review & editing. GL: Data curation, Investigation, Validation, Writing – review & editing. WL: Data curation, Visualization, Writing – review & editing. YZ: Formal analysis, Project administration, Writing – review & editing.
