# Supplementary material for: The impact of mixed planting of Poaceae species in the Qinghai-Tibet plateau region on forage yield, soil nutrients, and soil microbial communities
Source: Front Plant Sci. 2024 Apr 29;15:1370593. doi: 10.3389/fpls.2024.1370593 (PMC11089163; doi:10.3389/fpls.2024.1370593)
Supplement: Supplementary file 1 [file DataSheet_1.docx]

Supplementary Material

Table S1 Analysis of Variance (ANOVA) of Herbage Yield in Mixed Planting Artificial Grassland Academic

| Factor | Forage yield | |
| --- | --- | --- |
|  | F | *P* |
| Mixed planting artificial grassland | 780.955 | ＜0.001 |
| Planting year | 856.172 | ＜0.001 |
| Mixed artificial grassland ×Planting year | 132.825 | ＜0.001 |

Figure S1. Herbage Yields in Different Types of Artificial Grasslands

Note: Different letters indicate significant differences in grass yield among artificial grasslands of same growth ages(*P*<0.05).

Table S2 Analysis of Variance (ANOVA) for soil nutrient and soil enzyme activity in mixed-grassland.

| Index | F | P |
| --- | --- | --- |
| soil nitrate reductase（SNR） | 461.635 | >0.001 |
| soil alkaline phosphatase（SAP） | 182.206 | >0.001 |
| Soil urease（SU） | 17.082 | 0.003 |
| soil total nitrogen（STN） | 22.675 | 0.002 |
| soil total phosphorus（STP） | 23.786 | 0.001 |

Table S3. Soil microbial community alpha diversity.

|  |  | *Puccinellia* | *Poa+Puccinellia* | *Poa* | F | P |
| --- | --- | --- | --- | --- | --- | --- |
| Fungal | Sobs | 977.33±17.21 | 934±29.82 | 968±30.45 | 2.216 | 0.190 |
|  | ACE | 1077.82±17.44 | 1037.5±31.12 | 1099.74±31.74 | 256.850 | 0.081 |
|  | Shannon | 6.76±0.03b | 6.89±0.02a | 6.71±0.01b | 3.620 | >0.001 |
|  | Simpson | 0.98±0.01a | 0.98±0.01a | 0.97±0.01b | 3.934 | >0.001 |
|  | Goods coverage | 0.999±0.0001 | 0.999±0.0001 | 0.999±0.0001 | 2.493 | 0.163 |
| Bacterial | Sobs | 4323±41.94 | 4532.33±201.65 | 4306.33±74.27 | 2.978 | 0.126 |
|  | ACE | 4868.7±37.21 | 5087.97±191.78 | 4847.9±50.82 | 3.908 | 0.082 |
|  | Shannon | 9.63±0.04b | 9.67±0.03ab | 9.75±0.05a | 6.095 | 0.036 |
|  | Simpson | 0.99±0.01b | 0.99±0.01b | 0.99±0.01a | 20.938 | 0.002 |
|  | Goods coverage | 0.999±0.0001 | 0.999±0.0001 | 0.999±0.0001 | 2.369 | 0.152 |

Note: Data are expressed as mean ± SD. Different letters indicate significant differences. (*P*<0.05)

Table S4. Analysis of Variance (ANOVA) at the phylum level of soil microorganisms.

| Domain | Phylum | *Puccinellia* | *Poa+Puccinellia* | *Poa* | F | *P* |
| --- | --- | --- | --- | --- | --- | --- |
| Bacteria | *Proteobacteria* | 32.38±0.53 | 29.59±0.24 | 31.61±0.69 | 50.693 | 0.000 |
| Bacteria | *Acidobacteriota* | 15.15±0.52 | 18.13±0.2 | 17.15±0.22 | 146.167 | 0.000 |
| Bacteria | *Planctomycetota* | 11.81±0.33 | 12.06±0.14 | 11.33±0.24 | 15.275 | 0.004 |
| Bacteria | *Bacteroidota* | 10.12±0.3 | 9.26±0.19 | 8.56±0.76 | 97.073 | 0.000 |
| Bacteria | *Actinobacteriota* | 7.69±0.09 | 7.68±0.26 | 7.37±0.82 | 53.572 | 0.000 |
| Bacteria | *Gemmatimonadota* | 5.12±0.17 | 4.66±0.17 | 4.87±0.16 | 273.039 | 0.000 |
| Bacteria | *Chloroflexi* | 3.23±0.21 | 3.27±0.11 | 3.35±0.32 | 4.936 | 0.054 |
| Bacteria | *Verrucomicrobiota* | 2.46±0.08 | 3.41±0.07 | 2.81±0.23 | 9.968 | 0.012 |
| Bacteria | *Patescibacteria* | 1.99±0.12 | 2.07±0.01 | 2.08±0.13 | 4.081 | 0.076 |
| Bacteria | *Myxococcota* | 1.4±0.04 | 1.22±0.08 | 1.45±0.08 | 4.189 | 0.029 |
| Bacteria | *Firmicutes* | 0.59±0.04 | 0.68±0.05 | 0.7±0.02 | 3.810 | 0.003 |
| Bacteria | *Nitrospirota* | 0.56±0.02 | 0.4±0.01 | 0.8±0.03 | 9.968 | 0.000 |
| Fungi | *Ascomycota* | 71.66±0.21 | 69.48±1.07 | 74.94±0.39 | 50.693 | 0.000 |
| Fungi | *Mortierellomycota* | 10.04±0.44 | 16.63±0.7 | 10.71±0.35 | 146.167 | 0.000 |
| Fungi | *Basidiomycota* | 5.06±0.36 | 4.72±0.15 | 5.85±0.22 | 15.275 | 0.004 |
| Fungi | *Olpidiomycota* | 2.17±0.25 | 0.89±0.03 | 0.52±0.07 | 97.073 | 0.000 |
| Fungi | *Mucoromycota* | 0.16±0.06 | 0.57±0.07 | 0.16±0.02 | 53.572 | 0.000 |
| Viridiplantae | *Anthophyta* | 0.04±0.01 | 0.41±0.03 | 0.07±0.01 | 273.039 | 0.000 |
| Fungi | *Chytridiomycota* | 0.14±0.02 | 0.13±0.02 | 0.08±0.03 | 4.936 | 0.054 |
| Fungi | *Glomeromycota* | 0.06±0.01 | 0.03±0.01 | 0.07±0.02 | 9.968 | 0.012 |
| Fungi | *Zoopagomycota* | 0.03±0.02 | 0.04±0.00 | 0.02±0.00 | 4.081 | 0.076 |

Note: Data are expressed as mean ± SD.

**
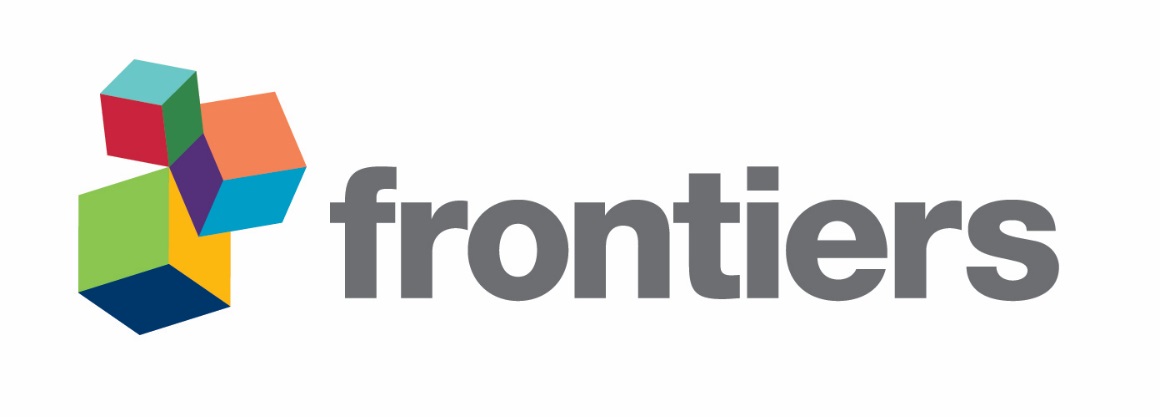
**
